# Pyrogallol from *Spirogyra neglecta* Inhibits Proliferation and Promotes Apoptosis in Castration-Resistant Prostate Cancer Cells via Modulating Akt/GSK-3*β*/*β*-catenin Signaling Pathway

**DOI:** 10.3390/ijms24076452

**Published:** 2023-03-29

**Authors:** Punnida Arjsri, Sariya Mapoung, Warathit Semmarath, Kamonwan Srisawad, Wirote Tuntiwechapikul, Supachai Yodkeeree, Pornngarm Dejkriengkraikul

**Affiliations:** 1 Department of Biochemistry, Faculty of Medicine, Chiang Mai University, Chiang Mai 50200, Thailand; 2 Anticarcinogenesis and Apoptosis Research Cluster, Faculty of Medicine, Chiang Mai University, Chiang Mai 50200, Thailand; 3 Center for Research and Development of Natural Products for Health, Chiang Mai University, Chiang Mai 50200, Thailand; 4 Akkraratchkumari Veterinary College, Walailak University, Nakhon Si Thammarat 80160, Thailand

**Keywords:** *Spirogyra neglecta*, pyrogallol, castration-resistant prostate cancer, anti-cancer properties, apoptosis induction, cancer cells proliferation, *β*-catenin signaling pathway

## Abstract

Castration-resistant prostate cancer (CRPC) is an advanced form of prostate cancer associated with poor survival rates. The high proliferation and metastasis rates have made CRPC one of the most challenging types of cancer for medical practitioners and researchers. In this study, the anti-cancer properties and inhibition of CRPC progression by *S. neglecta* extract and its active constituents were determined using two CRPC cell lines, DU145 and PC3. The ethyl acetate fraction of *S. neglecta* (SnEA) was obtained using a solvent-partitioned extraction technique. The active constituents of SnEA were then determined using the HPLC technique, which showed that SnEA mainly contained syringic acid, pyrogallol, and p-coumaric acid phenolic compounds. After the determination of cytotoxic properties using the SRB assay, it was found that pyrogallol, but not the other two major compounds of SnEA, displayed promising anti-cancer properties in both CRPC cell lines. SnEA and pyrogallol were then further investigated for their anti-proliferation and apoptotic induction properties using propidium iodide and Annexin V staining. The results showed that SnEA and pyrogallol inhibited both DU145 and PC3 cell proliferation by inducing cell cycle arrest in the G0/G1 phase and significantly decreased the expression of cell cycle regulator proteins (cyclin D1, cyclin E1, CDK-2, and CDK-4, *p* < 0.001). SnEA and pyrogallol treatments also promoted apoptosis in both types of CRPC cells through significantly downregulating anti-apoptotic proteins (survivin, Bcl-2, and Bcl-xl, *p* < 0.001) and upregulating apoptotic proteins (cleaved-caspase-9, cleaved-caspase-3 and cleaved-PARP-1, *p* < 0.001). Mechanistic study demonstrated that SnEA and pyrogallol inactivated the Akt signaling pathway leading to enhancement of the active form of GSK-3*β* in CRPC cell lines. Therefore, the phosphorylation of *β*-catenin was increased, which caused degradation of the protein, resulting in a downregulation of *β*-catenin (unphosphorylated form) transcriptional factor activity. The current results reflect the potential impact of *S. neglecta* extract and pyrogallol on the management of castration-resistant prostate cancer.

## 1. Introduction

Prostate cancer is one of the most prevalent forms of cancer among males worldwide. It can be categorized as benign if it grows slowly and is restricted to the prostate gland, where it is unlikely to cause significant harm. In the minority of patients whose cancers are aggressive or advanced, therapeutic options include prostatectomy, radiation therapy and, more commonly, androgen-deprivation therapy [1,2]. Castration-resistant prostate cancer (CRPC) is an advanced form of prostate cancer characterized by disease progression following surgical or pharmaceutical (androgen deprivation) castration. It is unknown how prostate cancer cells become castrate-resistant; however, it has been hypothesized that androgen ablation offers a selection advantage to androgen-independent cells, which proliferate and repopulate the tumor. This type of cancer displays aggressive characteristics which include a high proliferation rate and high metastasis potential, resulting in a poor prognosis with overall reduced survival for the patients [3,4,5,6].

Nowadays, CRPC remains a clinically challenging late-stage cancer with no curative treatment options. Taxanes, especially docetaxel and cabazitaxel, have first- and second-line indications for CRPC, respectively, with both providing a survival benefit [7]. However, recent reports of ineffective treatment in most of the patients and serious side effects have led to the search for alternative treatment options. In the molecular perspective of CRPC type prostate cancer, alterations of components in the PI3K/Akt/mTOR signaling pathway have been reported in 42% of primary prostate tumors and 100% of metastatic tumors from biopsy specimens [8,9]. These alterations lead to increased Akt signaling activity. The Akt pathway contributes to prostate cancer development and progression through interacting with other cell signaling pathways important for cellular survival, proliferation, and differentiation, including the GSK-3*β*/*β*-catenin signaling pathway [10,11].

Accordingly, the novel therapeutic approach for CRPC patients of targeting the Akt or *β*-catenin signaling pathway in prostate cancer cells could potentially improve the effectiveness of CRPC treatments [12]. Historically, naturally occurring compounds have played an essential role in the prevention and treatment of various type of cancers. The use of natural compounds as a combination therapy has the potential to be more effective than single-agent therapy [13,14,15]. Hence, the discovery of novel anti-cancer properties compounds from medicinal herbs has represented a significant step in developing an effective treatment for aggressive types of cancer such as CRPC.

*Spirogyra neglecta* (Hassall) Kützing or *Spirogyra neglecta* is a freshwater green alga widely found in freshwater locations in the north and northeast of Thailand. Previous data found that *S. neglecta* contains high amounts of phytochemicals such as phenolics, alkaloids, steroids, flavonoids, tannins and terpenoids [16,17,18]. Interestingly, many phenolic compounds have been identified in *S. neglecta* crude extracts such as gallic acid, catechin, pyrogallol, syringic acid, protocatechuic acid, and chlorogenic acid, and these phenolic compounds are reported to show high levels of antioxidant activity [19,20,21]. Furthermore, *S. neglecta* exhibits various beneficial biological properties including antioxidant, anti-bacterial, anti-inflammatory, and anti-diabetic activities [18,22,23,24]. Numerous studies have investigated the anti-cancer properties of *S. neglecta* extracts. For instance, the water extract of *S. neglecta* showed a protective effect against diethylnitrosamine-induced hepatocarcinogenesis in rats [25]. *S. neglecta* extract also inhibited the early stages of DMH-induced colon carcinogenesis in rats by modulating xenobiotic metabolizing enzymes, inhibition of cell proliferation, and induction of apoptosis [26]. In our previous study, we found that the ethyl acetate fraction of *S. neglecta* inhibited the metastasis of PC3 prostate cancer cells by inhibiting MMP-9 secretion, epithelial-mesenchymal transition, and the Akt signaling pathway. The plant-based metabolomic profiling approach revealed that gallic acid was a potential anti-metastasis compound in *S. neglecta* extract [27]. However, the inhibitory effects of *S. neglecta* and its active constituents on human prostate cancer cells proliferation and apoptosis have not yet been elucidated. Mechanistic studies on the anti-cancer properties of *S. neglecta* and its active compounds should be explored.

This study aims to investigate the in vitro anti-cancer properties of *S. neglecta* extract and its bioactive compounds on CRPC cell lines (DU145 and PC3 cells). DU145 and PC3 cells are commonly used as representative androgen non-responsive prostate cancer cell lines [28,29]. The cytotoxicity, anti-proliferation, apoptotic induction, and modulation of Akt and *β*-catenin signaling pathways by *S. neglecta* extract and its potential bioactive compounds on DU145 cells and PC3 cells were investigated. The findings obtained from this study could introduce *S. neglecta* extract and pyrogallol as potential candidates for adjuvant therapy in the treatment of castration-resistant prostate cancer patients.

## 2. Results

### 2.1. Extraction and Phenolic Compound Identification of S. neglecta Ethyl Acetate Fraction (SnEA)

After undergoing the ethanolic extraction process, *S. neglecta* ethanolic extract (SnEE, 21.00% yield) was obtained. To concentrate the phenolic content in *S. neglecta*, the ethanolic extract was further extracted through use of the solvent partition extraction technique. Accordingly, the *S. neglecta* ethyl acetate fraction (SnEA, 3.33% yield), *S. neglecta* dichloromethane fraction (SnDM, 2.16% yield), and *S. neglecta* residue fraction (Sn-H_2_O, 10.67% yield) were obtained. The total phenolic contents as determined by Folin–Ciocalteu assay of each fraction of the *S. neglecta* extracts are shown in Table 1. The phenolic contents were calculated using gallic acid as the gold standard solution [30,31]. The results indicate that SnEA contained significantly higher amounts of total phenolic content (735.42 ± 4.55 mg GA/g extract) when compared with SnEE (245.73 ± 6.58 mg GA/g extract), SnDM (37.68 ± 2.52 mg GA/g extract), and Sn-H_2_O (27.33 ± 0.99 mg GA/g extract). Therefore, the solvent-partitioned extraction technique could enrich the phenolic content, as seen in SnEA, and this fraction could then be further used to identify the existing bioactive phenolic compounds.

As a high amount of total phenolic content was observed in SnEA, we further identified the phenolic constituents using HPLC analysis. Eleven phenolic commercially available standards, including caffeic acid, catechin, chlorogenic acid, ellagic acid, ferulic acid, gallic acid, *p*-coumaric acid, protocatechuic acid, pyrogallol, syringic acid, and vanillic acid, were used to identify the phenolic constituents present in SnEA. The results indicated that syringic acid, pyrogallol, *p*-coumaric acid, catechin, and gallic acid were identified in SnEA. Syringic acid was found to be the major compound in SnEA at a concentration of 122.28 ± 11.72 mg/g extract, followed by pyrogallol, *p*-coumaric acid, catechin, and gallic acid, which were found at 107.53 ± 4.48 mg/g extract, 93.22 ± 8.23 mg/g extract, 10.11 ± 2.13 mg/g extract, and 9.96 ± 2.80 mg/g extract, respectively. However, caffeic acid, chlorogenic acid, ellagic acid, ferulic acid, protocatechuic acid, and vanillic acid were not detected in SnEA, as is shown in Table 2. Therefore, it can be concluded that the SnEA obtained from *S. neglecta* using the solvent-partitioned extraction technique contained three major phenolic compounds, namely, syringic acid, pyrogallol, and *p*-coumaric acid. These compounds, together with SnEA, were then used in further experiments in investigations on the relevant anti-cancer properties against CRPC cells.

### 2.2. Effect of SnEA and Its Major Phenolic Compounds on DU145 and PC3 Cells Viability

**Figure 1:**
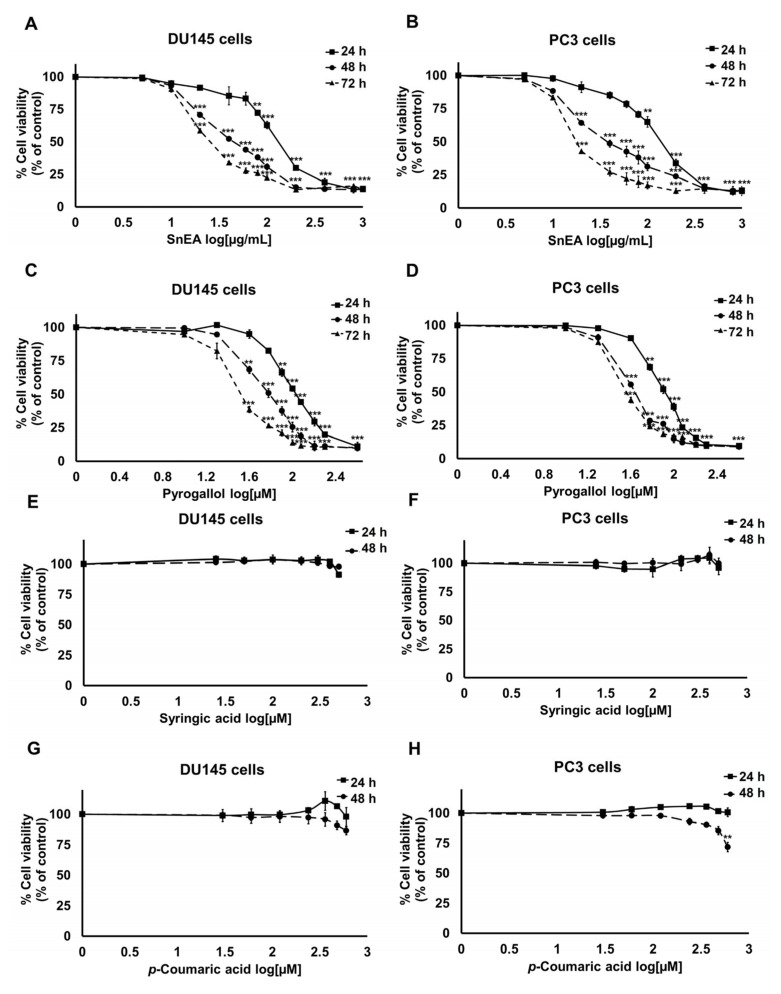
The effects of SnEA and its major compounds on DU145 and PC3 cells’ viability were determined by SRB assay and represented using a semi-log scale curve. The DU145 and PC3 cells were treated with 0–1000 µg/mL of SnEA (**A**,**B**) or pyrogallol at 0–200 µM (**C**,**D**) for 24, 48, and 72 h. DU145 and PC3 cells were treated with 0–500 µM syringic acid (**E**,**F**) and 0–600 µM *p*-coumaric acid (**G**,**H**) for 24 and 48 h. Data are presented as mean ± S.D. values of three independent experiments. ** *p* < 0.01 and *** *p* < 0.001 vs. the control (0 µg/mL or 0 µM).

To study the anti-cancer properties of *S. neglecta* extract on CRPC cells, the effects of SnEA and its major phenolic compounds—syringic acid, pyrogallol, and *p*-coumaric acid—on DU145 and PC3 cells in terms of cytotoxicity were determined using the SRB assay. After 48 h of incubation, the results showed that SnEA had significant cytotoxic effects on DU145 and PC3 cells, with 50% inhibitory concentrations (IC_50_) of 47.94 ± 0.70 and 42.97 ± 4.24 µg/mL, respectively (Figure 1A,B). Among the major compounds found in SnEA, pyrogallol demonstrated remarkable cytotoxicity on both DU145 and PC3 cells, with IC_50_ values of 58.89 ± 3.06 and 45.79 ± 1.82 µM, respectively (Figure 1C,D). In contrast, after 48 h of incubation, syringic acid and *p*-coumaric acid did not show any cytotoxic effects on either DU145 or PC3 cells (Figure 1E–H). From the results, it can be concluded that pyrogallol was the main compound in SnEA which might be bioactive and play an important role in anti-cancer properties. SnEA and pyrogallol were selected for further investigation of their anti-cancer properties on CRPC cells.

### 2.3. Evaluation of SnEA and Pyrogallol-Induced Toxicity in Normal Cells

**Figure 2:**
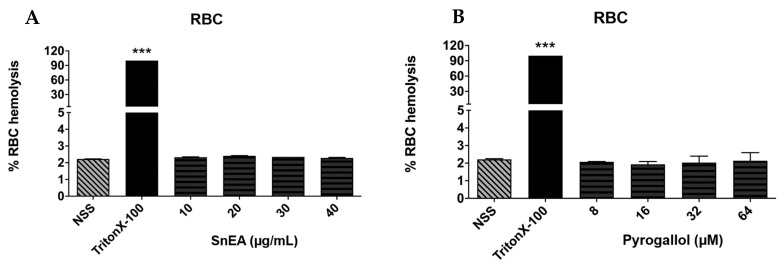
The effect of SnEA and pyrogallol on normal red blood cell (RBC) hemolysis. Red blood cells were treated with normal saline solution (NSS) (negative control), 1% TritonX-100 (positive control), 0–40 µg/mL of SnEA (**A**,**B**) and 0–64 µM of pyrogallol for 4 h at 37 °C. Data are presented as mean ± S.D. values of three independent experiments. *** *p* < 0.001 vs. TritonX-100 (positive control).

Phenolics are naturally occurring compounds that can be found in many plants and have been recognized as environment-friendly and safe. However, the degree of cytotoxicity of SnEA and the pyrogallol phenolic compound towards normal cells still needed to be confirmed. We determined the cytotoxicity of SnEA and pyrogallol on normal human dermal fibroblast (HDF) cells. The results indicate that after 48 h of incubation, SnEA and pyrogallol exhibited cytotoxicity effects on HDF cells with IC_50_ of 122.63 ± 2.25 µg/mL and 201.68 ± 28.72 µM, respectively. As shown in Table 3, SnEA has selectivity towards DU145 and PC3 cells with a selectivity index (SI) of 2.55 and 2.85, respectively. Meanwhile, pyrogallol had a selectivity index of more than three on both DU145 and PC3 cells. Accordingly, SnEA, as well as pyrogallol, exhibited high selectivity towards the CRPC type of prostate cancer. Moreover, SnEA at concentrations of 0–40 µg/mL, and pyrogallol at concentrations of 0–64 µM, did not induce red blood cell (RBC) hemolysis, as is shown in Figure 2. Thus, SnEA and pyrogallol demonstrated no harmful effects on normal cells at the concentrations used in our experimental studies.

### 2.4. Effects of SnEA and Pyrogallol on Cell Cycle Arrest in CRPC Cells

**Figure 3:**
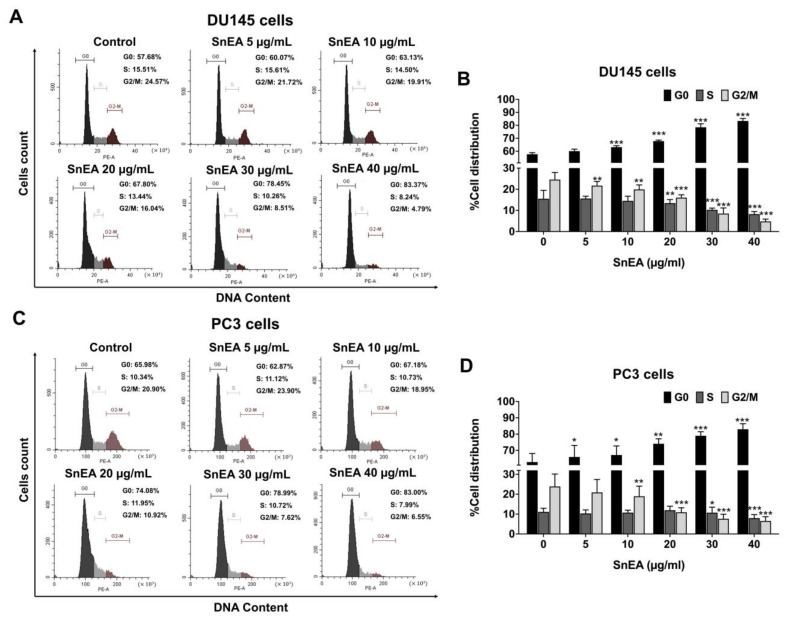
The effect of SnEA on cell cycle distribution in DU145 and PC3 cells. The DU145 (**A**,**B**) and PC3 (**C**,**D**) cells were treated with 0–40 µg/mL of SnEA for 24 h before being stained with PI dye. Cell cycle distribution was analyzed by flow cytometry (**A**,**C**). Bar graphs show the percentages of cells in the G0, S and G2/M phase (**B**,**D**). Data are presented as mean ± S.D. values of three independent experiments. * *p* < 0.05, ** *p* < 0.01 and *** *p* < 0.001 vs. the control (0 µg/mL).

The inhibitory effect of SnEA and its active compound, pyrogallol, on the cell viability of CRPC cells may affect two key cellular mechanisms, i.e., cell cycle arrest, and induction of apoptosis. To determine the effects of SnEA and pyrogallol on DU145 and PC3 cell proliferation, the distribution of cells in different phases was examined using PI staining and flow cytometry. After SnEA treatment, the number of G0/G1 phase profiles for both DU145 (Figure 3A,B) and PC3 (Figure 3C,D) cells was significantly increased in a dose-dependent manner (*p* < 0.001), whereas the number of cells in the S phase and the G2/M phase was significantly decreased in a dose-dependent manner (*p* < 0.001), as is shown in Figure 3.

**Figure 4:**
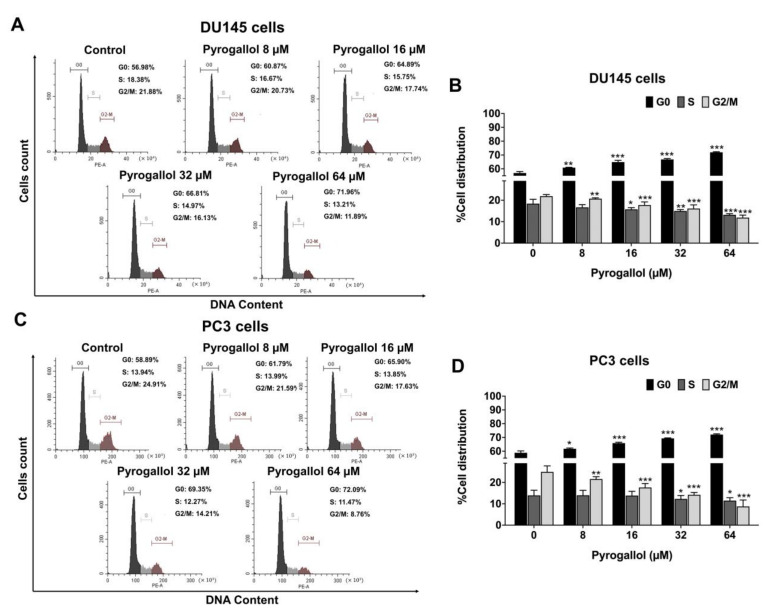
The effects of pyrogallol on cell cycle distribution in DU145 and PC3 cells. The DU145 (**A**,**B**) and PC3 (**C**,**D**) cells were treated with 0–64 µM of pyrogallol for 24 h before being stained with PI dye. Cell cycle distribution was analyzed by flow cytometry (**A**,**C**). The bar graphs show the percentages of cells in G0, S, and G2/M phases (**B**,**D**). Data are presented as mean ± S.D. values of three independent experiments. * *p* < 0.05, ** *p* < 0.01 and *** *p* < 0.001 vs. the control (0 µM).

Furthermore, the percentages of both DU145 (Figure 4A,B) and PC3 (Figure 4C,D) cells in the G0/G1 phase after pyrogallol treatment were significantly increased in a dose-dependent manner (*p* < 0.001), while the percentages in the S and G2/M phase were significantly decreased in a dose-dependent manner (*p* < 0.001), as shown in Figure 4. The results suggest that SnEA and its bioactive compound, pyrogallol, can cause CRPC cell cycle arrest at the G0/G1 phase.

### 2.5. Effects of SnEA and Pyrogallol on Cell Cycle-Regulated Proteins’ Expression in CRPC Cells

**Figure 5:**
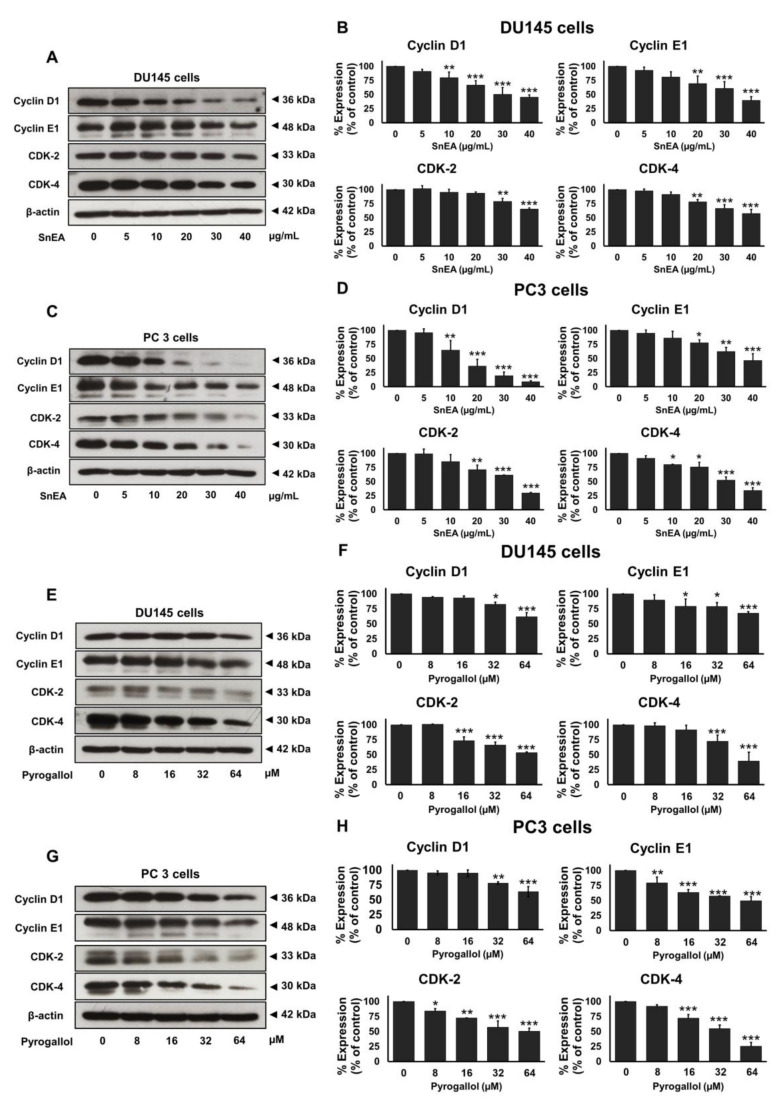
The effects of SnEA and pyrogallol on cell cycle regulator protein (Cyclin D1, Cyclin E1, CDK-2, and CDK-4) expression. DU145 (**A**,**B**) and PC3 (**C**,**D**) cells were treated with 0–40 µg/mL of SnEA for 24 h before being harvested. The DU145 (**E**,**F**) and PC3 (**G**,**H**) cells were treated with 0–64 µM of pyrogallol for 24 h before being harvested. The protein expression was determined by Western blot analysis. The band density was measured using Image J 1.410 software. Non-treatment of DU145 or PC3 cells are presented as 100% of the control. Data are presented as mean ± S.D. values of three independent experiments. * *p* < 0.05, ** *p* < 0.01 and *** *p* < 0.001 vs. the control (0 µg/mL or 0 µM).

To elucidate the mechanism by which SnEA and its bioactive compound, pyrogallol, regulate cell cycle progression in CRPC cells, Western blotting analysis was performed to measure the expression of cell cycle regulator proteins. The results indicate that SnEA markedly reduced cyclin D1, cyclin E1, CDK-2, and CDK-4 expression in both DU145 (Figure 5A,B) and PC3 (Figure 5C,D) cells in a dose-dependent manner (*p* < 0.001). Pyrogallol also markedly down-regulated cyclin D1, cyclin E1, CDK-2, and CDK-4 expression in both DU145 (Figure 5E,F) and PC3 (Figure 5G,H) cells in a dose-dependent manner (*p* < 0.001). These results demonstrate that SnEA, along with its active compound, pyrogallol, could induce cell cycle arrest and inhibit cell proliferation in both DU145 and PC3 cells at the G0/G1 phase via the inhibition of cyclin D1, cyclin E1, CDK-2, and CDK-4 protein expression.

### 2.6. Effects of SnEA and Pyrogallol on CRPC Cells Apoptosis

**Figure 6:**
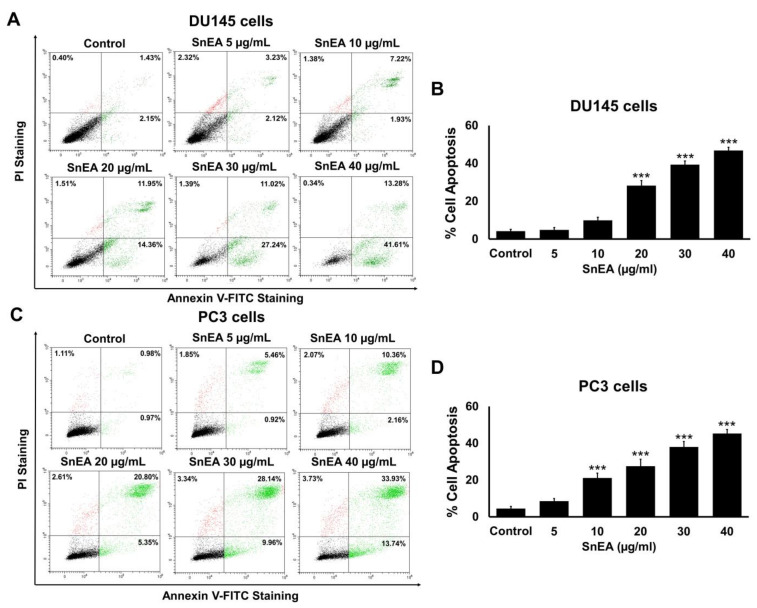
The effect of SnEA on apoptosis of DU145 and PC3 cells. The DU145 (**A**,**B**) and PC3 (**C**,**D**) cells were treated with 0–40 µg/mL of SnEA for 48 h before being co-stained with Annexin V-FITC and PI dyes. Cell death was analyzed by flow cytometry (**A**,**C**). The bar graphs show the percentages of apoptotic cells (**B**,**D**). Data are presented as mean ± S.D. values of three independent experiments. *** *p* < 0.001 vs. the control (0 µg/mL).

**Figure 7:**
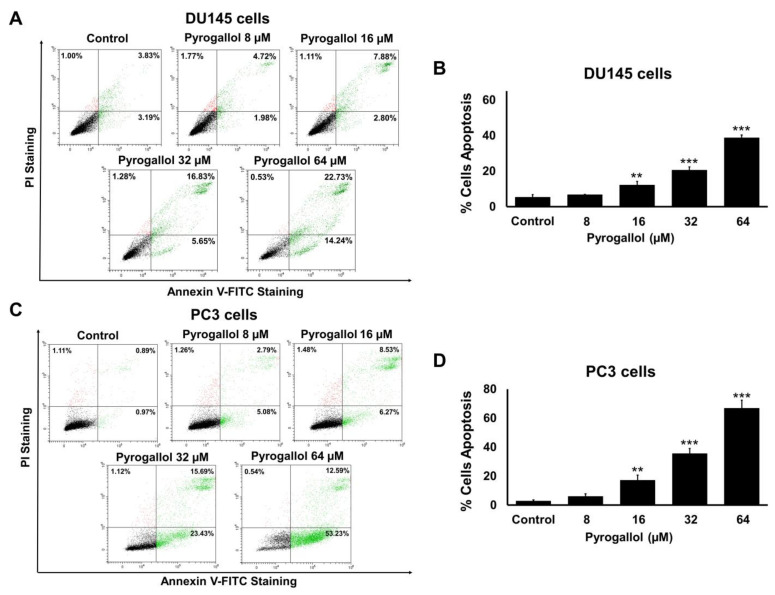
The effect of pyrogallol on apoptosis of DU145 and PC3 cells. The DU145 (**A**,**B**) and PC3 (**C**,**D**) cells were treated with 0–64 µM of pyrogallol for 48 h before being co-stained with Annexin V-FITC and PI dyes. Cell death was analyzed by flow cytometry (**A**,**C**). The bar graphs show the percentages of apoptotic cells (**B**,**D**). Data are presented as mean ± S.D. values of three independent experiments. ** *p* < 0.01 and *** *p* < 0.001 vs. the control (0 µM).

In this study, we tested whether SnEA and pyrogallol could induce apoptosis in human prostate cancer DU145 and PC3 cells by performing Annexin V-FITC and PI staining assays. DU145 and PC3 cells were treated with and without 5, 10, 20, 30, and 40 μg/mL SnEA or 8, 16, 32, and 64 μM pyrogallol for 24 h (Figures S1 and S2) and 48 h (Figure 6 and Figure 7). The results indicate that apoptotic cell accumulations were significantly enhanced by the SnEA treatment in a dose-dependent manner (*p* < 0.001), as is shown in Figure 6. Moreover, pyrogallol also significantly increased apoptotic cell populations in a dose-dependent manner for both DU145 and PC3 cells (*p* < 0.001), as is shown in Figure 7. These results clearly indicate that SnEA and pyrogallol could induce apoptosis in CRPC cells.

### 2.7. Effects of SnEA and Pyrogallol on Pro- and Anti-Apoptosis Protein Expression in CRPC Cells

**Figure 8:**
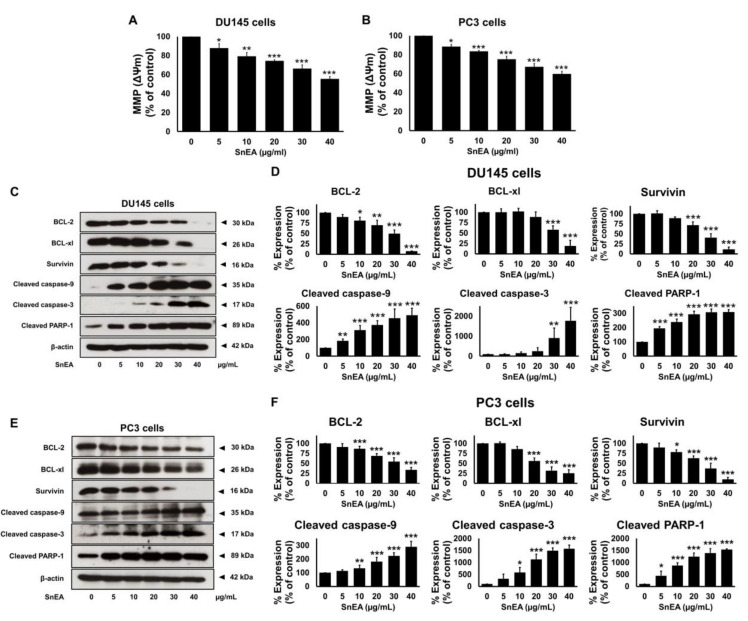
The effects of SnEA on DU145 and PC3 cells on the intrinsic apoptosis cascade. The DU145 and PC3 cells were treated with 0–40 µg/mL of SnEA for 48 h before being harvested. After treatment, the mitochondrial membrane potential (MMP(ΔΨm)) of DU145 (**A**) and PC3 (**B**) cells was determined using JC-10 dye staining. Pro-apoptotic (survivin, Bcl-2, and Bcl-xl) and anti-apoptotic (cleaved-caspase-9, cleaved-caspase-3, and cleaved-PARP-1) protein expressions of DU145 (**C**,**D**) and PC3 (**E**,**F**) cells were determined by Western blot analysis. The band density was measured using Image J software. Non-treatment of DU145 or PC3 cells is presented as 100% of the control. Data are presented as mean ± S.D. values of three independent experiments. * *p* < 0.05, ** *p* < 0.01 and *** *p* < 0.001 vs. the control (0 µg/mL).

**Figure 9:**
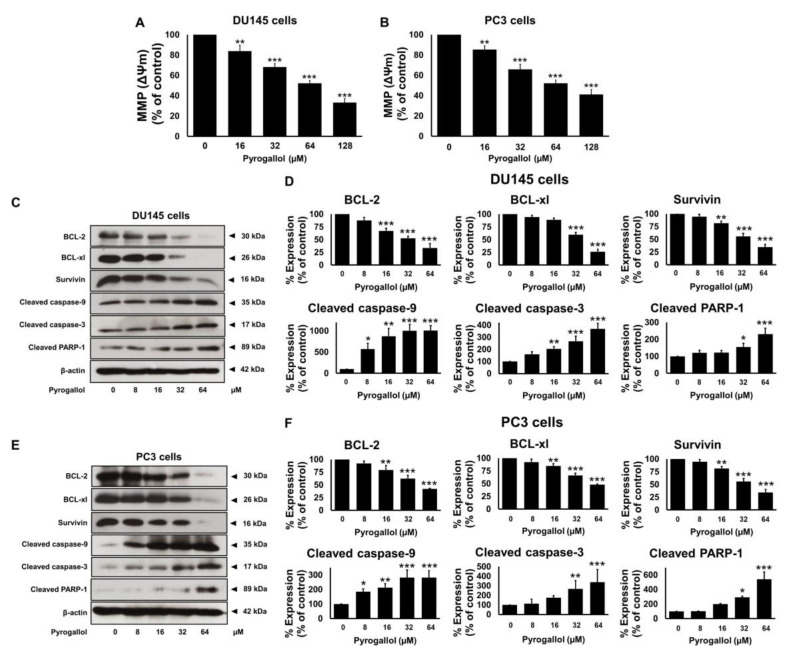
The effects of pyrogallol on DU145 and PC3 cells on the intrinsic apoptosis cascade. The DU145 and PC3 cells were treated with 0–64 µM of pyrogallol for 48 h before being harvested. After the treatment, the mitochondrial membrane potential (MMP(ΔΨm)) of DU145 (**A**) and PC3 (**B**) cells were determined using JC-10 dye staining. Pro-apoptotic (survivin, Bcl-2, and Bcl-xl) and anti-apoptotic (cleaved-caspase-9, cleaved-caspase-3 and cleaved-PARP-1) protein expressions of DU145 (**C**,**D**) and PC3 (**E**,**F**) cells were determined by Western blot analysis. The band density was measured using Image J software. Non-treatment of DU145 or PC3 cells is presented as 100% of the control. Data are presented as mean ± S.D. values of three independent experiments. * *p* < 0.05, ** *p* < 0.01 and *** *p* < 0.001 vs. the control (0 µg/mL).

To determine whether SnEA and pyrogallol could effectively induce apoptosis through caspase cascades in CRPC cells, the mitochondrial membrane potential (MMP, ΔΨm) was studied using JC-10 dye staining. Furthermore, the protein expression levels of the anti-apoptotic proteins, survivin, Bcl-xl, Bcl-2, along with pro-apoptotic proteins (cleaved-caspases-3, cleaved-caspases-9, cleaved-PARP-1), were determined using Western blot analysis. Mitochondrial depolarization was specifically indicated by JC-10 dye, which is a cationic dye that exhibits potential-dependent accumulation in mitochondria, as indicated by a fluorescence emission shift from red to green as the mitochondrial membrane loses its potential due to its damaged membrane. In this study, the SnEA-treated cells showed a significant decrease of ΔΨm when compared with the control (untreated cells) (*p* < 0.001) in both the DU145 and PC3 cell lines (Figure 8A,B). A decrease in ΔΨm led to activation of the intrinsic apoptosis signaling cascade. Western blotting analysis indicated that the SnEA treatment downregulated survivin, Bcl-xl, and Bcl-2 expression and activated cleaved-caspases-9, cleaved-caspases-3, and cleaved-PARP-1 in a dose-dependent manner (*p* < 0.001) in DU145 (Figure 8C,D) and PC3 cells (Figure 8E,F). Pyrogallol also decreased the ΔΨm in both DU145 and PC3 cells as shown in Figure 9A,B, respectively. Moreover, the effect of pyrogallol on the intrinsic apoptosis signaling cascade indicated that pyrogallol-treated cells significantly decreased the expression of anti-apoptotic proteins, survivin, Bcl-xl, and Bcl-2, while significantly increasing cleaved-caspases-9, cleaved-caspases-3, and cleaved-PARP-1 protein expression in a dose-dependent manner (*p* < 0.001) in DU145 (Figure 9C,D) and PC3 cells (Figure 9E,F). Overall, it can be concluded that SnEA and pyrogallol were potentially responsible for the anti-cancer action seen in both types of CRPC cells, via disruption of the ΔΨm and decreased expression of anti-apoptotic proteins (survivin, Bcl-xl, and Bcl-2) leading to activation of the intrinsic apoptosis signaling cascade (caspases-9, caspases-3, and PARP-1).

### 2.8. Effects of SnEA and Pyrogallol on the Akt/GSK-3β/β-Catenin Signaling Pathway in PC3 Cells

Previous studies have reported that in androgen-independent prostate cancer, the cancer exhibits high activity of the PI3K/Akt signaling pathway. Normally, the phosphorylated form of the Akt protein induces the phosphorylation of GSK-3*β* at the ser9 site to inactivate the activity of GSK-3*β*, resulting in a decrease in the *β*-catenin phosphorylation via the GSK-3*β* protein. Subsequently, *β*-catenin can be phosphorylated by GSK-3*β*, and the phosphorylated form of *β*-catenin can then be attached to ubiquitin and degraded by the proteasome. In the event that the phosphorylation of *β*-catenin is blocked, the unphosphorylated form of *β*-catenin can translocate to the nucleus and regulate the expression of target genes [32,33].

**Figure 10:**
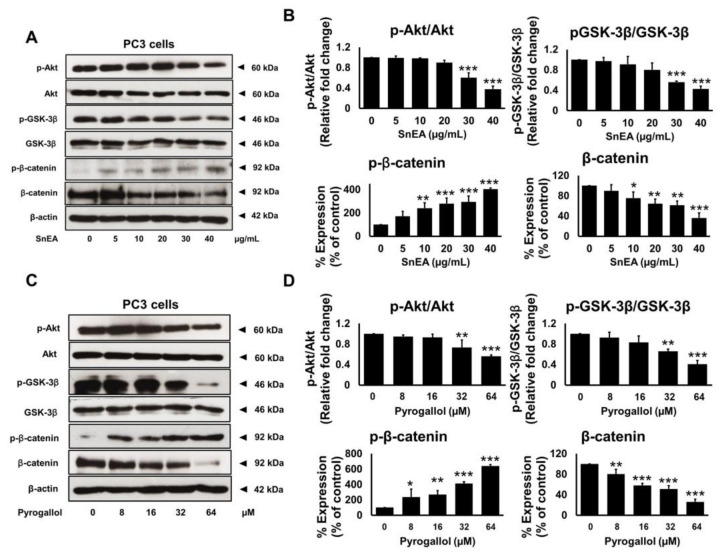
The effects of SnEA and pyrogallol on the Akt/GSK-3*β*/*β*-catenin signaling pathway modulation in PC3 cells. PC3 cells were treated with 0–40 µg/mL of SnEA (**A**,**B**) or 0–64 µM of pyrogallol (**C**,**D**) for 24 h before being harvested. The phosphorylation of Akt, GSK-3*β*, and *β*-catenin were determined by Western blot analysis. Band density was measured using Image J software. Non-treatment of DU145 or PC3 cells is presented as 100% of the control. Data are presented as mean ± S.D. values of three independent experiments. * *p* < 0.05, ** *p* < 0.01 and *** *p* < 0.001 vs. the control (0 µg/mL or 0 µM).

To examine the upstream regulatory pathway, which is responsible for the anti-cancer properties of SnEA and pyrogallol in CRPC cells, we determined the inhibitory effects of SnEA and pyrogallol on the protein expression levels in the Akt/GSK-3*β*/*β*-catenin signaling pathway. The cellular effects on the apoptosis proteins and cell cycle-regulated proteins of SnEA and pyrogallol in DU145 and PC3 cells were similar, as observed in Section 2.5 and Section 2.7. Therefore, we selected the PC3 cells as the representative for the mechanistic investigation in this section. The protein expressions of the Akt/GSK-3*β*/*β*-catenin signaling pathway in PC3 cells were examined using Western blot analysis. The results indicated that the phosphorylation of the Akt and GSK-3*β* proteins was significantly decreased, whereas the phosphorylation of *β*-catenin significantly increased, in both SnEA (Figure 10A,B) and pyrogallol-treated PC3 cells (Figure 10C,D, *p* < 0.001). The amount of the unphosphorylated form of *β*-catenin (active form) decreased significantly in a dose-dependent manner (*p* < 0.001) in both SnEA and pyrogallol treated PC3 cells, as is shown in Figure 10. Overall, from our results, it can be inferred that treatment with SnEA or its active compound, pyrogallol, could attenuate the Akt/GSK-3*β*/*β*-catenin signaling pathway resulting in downregulation of the cell cycle-regulated proteins and anti-apoptotic protein expression in CRPC cells.

## 3. Discussion

Castration-resistant prostate cancer (CRPC) is a specific type of cancer with poor prognosis and impaired quality of life. Approximately 10–20% of patients with prostate cancer develop the castration-resistant stage during androgen deprivation therapy, within 5 years of follow-up [34,35]. Furthermore, the CRPC type of prostate cancer usually progresses quickly with a high proliferation rate and high metastasis rate, resulting in a survival of less than 10 years in CRPC patients [4,6]. In this study, we attempted to search for a potential therapeutic or adjuvant agent for CRPC. We found that pyrogallol from freshwater algae, *S. neglecta*, possesses anti-cancer properties, as evidenced through its inhibition of prostate cancer cell proliferation and promotion of apoptosis in two CRPC cell lines, DU145 and PC3.

*S. neglecta* alga was previously reported for its antioxidant, anti-bacterial and anti-inflammatory properties [22,23,36,37]. Regarding anti-cancer properties, *S. neglecta* water extract demonstrated in vivo cancer chemopreventive effects on DEN-induced hepatocarcinogenesis [25] as well as on the management of early stages of colon carcinogenesis in rat models [26]. In our previous study, using the plant-based metabolomic profiling technique, the *S. neglecta* ethyl acetate fraction inhibited PC3 prostate cancer cell metastasis via the inhibition of Akt/MAPK signaling pathway [27]. In this study, we further investigated the anti-cancer properties of *S. neglecta* ethyl acetate extract on CRPC cells (DU145 and PC3 cells). The selection of PC3 and DU145 cell lines was based on their aggressiveness and androgen hormone responsiveness. These cell lines are castration-resistant prostate cancer cells that lack androgen receptors and are not influenced by androgen hormone [38,39]. They are commonly used as CRPC cell lines and exhibit remarkable characteristics, such as being androgen-independent and proliferation in normal medium with FBS [40]. Nonetheless, it should be noted that although CRPC cell lines can survive androgen-deprivation conditions, the efficacy of SnEA or pyrogallol may vary in comparison to cells cultured with FBS. Therefore, as our study focuses on the inhibition of castration-resistant prostate cancer cells progression by *S. neglecta* extract and its active compound, these two cell lines are the best option to represent the aggressiveness or the progression of prostatic adenocarcinoma in our investigation. Herein, our findings demonstrated that SnEA displayed anti-cancer properties through the inhibition of cancer cells proliferation and apoptosis induction. Mechanistically, this was via the Akt/GSK-3*β*/*β*-catenin pathway in the DU145 and PC3 cell lines.

*S. neglecta* was previously acknowledged for its high phenolic contents. In this study, the solvent-partitioned technique was used to obtain the ethyl acetate fraction of *S. neglecta* (SnEA). SnEA contains a very high amount of total phenolic content, in which pyrogallol, syringic acid, and *p*-coumaric acid predominate. According to the anti-cancer properties screening by SRB assay, only pyrogallol demonstrated a potential cytotoxic effect on prostate cancer cell lines (both DU145 and PC3 cells), while syringic acid, and *p*-coumaric acid showed no cytotoxicity on these cell lines. Regarding the other minor phenolic compounds found in SnEA (catechin and gallic acid), the results from the SRB assay revealed that after 48 h of incubation, catechin had no cytotoxicity effect on either DU145 or PC3 cells at the concentrations of 0–100 µg/mL. Likewise, gallic acid showed much less cytotoxicity on both DU145 and PC3 cells when compared with pyrogallol. For this reason, pyrogallol was chosen as the potential active compound and was further investigated along with SnEA for anti-cancer properties in CRPC cell lines. To investigate the anti-cancer properties, a concentration range of pyrogallol was selected based on the concentrations that were determined by HPLC analysis. Therefore, in our experiments, a concentration of 0–64 μM of pyrogallol was the representative amount (as identified in SnEA at a concentration of 0–40 μg/mL).

Pyrogallol, the bioactive compound in *S. neglecta*, is a ubiquitous phenolic moiety found in a variety of flavonoids and polyphenols in edible plants, such as cacao, nuts, fruit peels, vegetables, and medicinal plants. A previous literature review found that pyrogallol could regulate the expression of pro-inflammatory genes in bronchial epithelial cells [41]. In terms of anti-cancer properties, pyrogallol could induce cell cycle arrest in Calu-6 human lung cancer cells [42] and induce apoptosis in SNU-484 gastric cancer cells [43]. Furthermore, pyrogallol was identified as an active component of *Emblica officinalis* extracts and possessed anti-proliferative effects on human cancer cells such as K562, Jurkat, Raji, Hep3B, Huh7, and HELA cells [44,45,46]. It was previously reported that pyrogallol could act as a reducing agent by auto-oxidizing and forming a free radical under alkaline conditions [47]. Regarding the impact of pyrogallol on normal cells in our study, we performed cytotoxicity testing on normal cells such as human dermal fibroblasts and red blood cells. The results displayed selectivity towards prostate cancer cells (PC3 and DU145) with a high selectivity index (SI) of pyrogallol and SnEA, indicating their lesser harmful effects on normal cells.

In this study, it was found that SnEA and pyrogallol possessed strong anti-cancer properties against DU145 and PC3 cells. SnEA and pyrogallol could induce cell cycle arrest in G0/G1 phase and significantly decrease the expression of the cell cycle regulator proteins, cyclin D1, cyclin E1, CDK-2, and CDK-4. It is well documented that cell proliferation is regulated by several cyclins and cyclin-dependent kinases (CDKs) [48,49]. Therefore, natural compounds that could regulate the cell cycle are regarded as a promising candidate for cancer therapy [50]. Herein, we showed that SnEA and pyrogallol induced G0/G1 arrest in DU145 and PC3 cells. The activation of a CDK requires binding to a specific regulatory subunit, called a cyclin. Specifically, at the G0/G1 phase of the cell cycle, cyclin D1/CDK-4 and cyclin E1/CDK-2 complexes are required for the initiation of the DNA replication process. In our study, it was found that SnEA and pyrogallol downregulated cyclin D1, cyclin E1, CDK-2, and CDK-4 protein expression. This indicates that SnEA and pyrogallol induced CRPC cell cycle arrest at the G0/G1 phase as a result of the inhibition of cell cycle-regulated proteins at this phase.

Apoptosis or programmed cell death is a process of cell suicide through intracellular cell death mechanisms [51]. In this study, we found that SnEA and pyrogallol could promote apoptosis in both types of CRPC cells through the downregulation of the anti-apoptotic proteins (survivin, Bcl-2, and Bcl-xl) and the upregulation of the apoptotic proteins (cleaved-caspase-9, cleaved-caspase-3, and cleaved-PARP-1). Moreover, SnEA and pyrogallol could drive the CRPC cells’ apoptosis via the intrinsic apoptosis pathway. The intrinsic pathway, or mitochondrial pathway, is activated by intracellular signals that are generated when the mitochondrial membrane loses its potential (ΔΨm). Then, cytochrome c is released from the mitochondrial intermembrane space to the cytosol, which activates the caspase enzymes cascade, leading to apoptosis [52,53]. According to our results, SnEA and pyrogallol disrupted the mitochondrial membrane potential (ΔΨm) in both types of CRPC cell line. Furthermore, SnEA and pyrogallol downregulated protein expression of survivin, Bcl-2, and Bcl-xl, which are anti-apoptotic proteins, and activated the caspase-9/caspase-3/PARP-1 cascade in both types of CRPC cells. Therefore, it can be inferred that SnEA and pyrogallol could exert their anti-cancer properties potentially through the regulation of the cell cycle and induction of the intrinsic apoptosis pathway in CRPC cells.

Currently, targeted cancer therapy is being investigated for a better understanding of the mechanisms related to the development and progression of CRPC [3,54]. Identifying an effective therapeutic target for CRPC treatment is crucial. Studies have shown that alteration and over-activation of the PI3K/Akt signaling pathway are prevalent in prostate cancer patients, particularly in CRPC cells [55,56]. Additionally, an aberrant *β*-catenin pathway plays a pivotal role in various cancers including prostate cancer. High *β*-catenin levels increase the transcription of genes involved in cell proliferation, including CCND1, c-myc, and c-jun. The PI3K/Akt cascade interacts with multiple co-operative signal transduction molecules, and one of them is the activation of *β*-catenin transcription factor [10,11]. Notably, phosphorylation of GSK-3*β* at Ser9 by phosphorylated Akt (p-Akt) induces GSK-3*β* inactivation and inhibits its ability to promote the degradation of *β*-catenin. Studies have shown that overactivation of the Akt signaling pathway could contribute to CRPC development [57,58]. As a result, the Akt/GSK-3*β*/*β*-catenin signaling pathway has been indicated as an important therapeutic target for drug design to inhibit progression in many types of cancer cells [59,60,61]. Previous studies have shown the potential of targeting the Akt/GSK-3*β*/*β*-catenin signaling pathway to inhibit cancer cells progression. Toosendanin, a triterpenoid extracted from the bark or fruits of *Melia toosendan* Sieb et Zucc inhibited growth and induced apoptosis in colorectal cancer through suppression of the Akt/GSK-3*β*/*β*-catenin pathway in vitro and in vivo [62]. Astragaloside IV, an active ingredient in *Astragalus membranaceus*, inhibited non-small cell lung cancer development via inhibition of the Akt/GSK-3*β*/*β*-catenin signaling axis, resulting in the induction of apoptosis [63]. Therefore, we hypothesized that SnEA and pyrogallol could inhibit CRPC proliferation and induce CRPC cell apoptosis by inhibiting the Akt/GSK-3*β*/*β*-catenin signaling pathway and inhibiting the expression of cell cycle-regulated proteins and anti-apoptosis proteins. Our results showed that SnEA and pyrogallol inhibited the phosphorylation of Akt and GSK-3*β* and increased the phosphorylation of *β*-catenin. This could lead to increased degradation of *β*-catenin, resulting in decreased level of unphosphorylated *β*-catenin (active form) in the cell lysate. To gain a more precise understanding of the specific steps in the inhibition by *S. neglecta* extract and pyrogallol observed in CRPC cells, it may be beneficial to utilize inhibitors involved in the Akt/GSK-3*β*/*β*-catenin signaling pathway, such as AZD8835 (PI3K inhibitor) [64], GSK-690693 (AKT/GSK inhibitor) [65], or PRI-724 (*β*-catenin inhibitor) [66]. Furthermore, it is worth investigating the PI3K/AKT and Wnt/*β*-catenin pathways separately, with more upstream/downstream proteins being evaluated. This could be a limitation of our current study.

**Figure 11:**
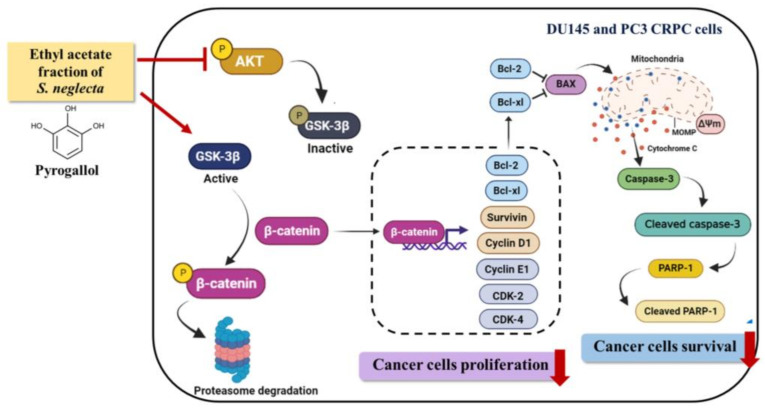
Schematic mechanism of how the ethyl acetate fraction of *S. neglecta* (SnEA) and pyrogallol downregulate cell cycle-regulated protein and anti-apoptotic protein expressions leading to cell cycle arrest at the G0/G1 phase and apoptosis via regulation of the Akt/GSK-3*β*/*β*-catenin pathway in CRPC cells.

In conclusion, the current study showed that SnEA and pyrogallol inhibited the expression of cyclin D1 and survivin, which are responsive proteins of *β*-catenin transcription factor, resulting in cell cycle arrest at G0/G1 phase and CRPC cell apoptosis. Figure 11 illustrates the mechanisms by which SnEA and pyrogallol inhibit CRPC cell proliferation and induce apoptosis. This study demonstrates that a phenolic-containing plant has anti-cancer effects on CRPC cells by inducing apoptosis, regulating cell cycle proteins, and targeting Akt and β-catenin signaling pathways. However, further investigation is needed to determine how pyrogallol inhibits the Akt/GSK-3β/β-catenin pathway. It is unclear whether the compound directly binds to Akt, GSK-3β, β-catenin, or upstream proteins, and specific protein targets require identification. Additional molecular-level studies and molecular docking would provide valuable insight into the mechanisms of action of SnEA and pyrogallol in inhibiting the progression of CRPC cells. Further investigation on non-CRPC cell lines, such as LNCaP, would help to determine the specificity and the potential for broader clinical application of SnEA and pyrogallol. It is essential to conduct additional research on the anti-cancer properties of *S. neglecta* and, in particular, pyrogallol in animal models. Further studies, including acute toxicity testing, assessing the stability of the compound and studying its pharmacokinetics, should be performed to validate its anti-cancer activities and efficacy before clinical trials can proceed. Overall, the information data obtained from this study may lead to improving the management of castration-resistant prostate cancer.

## 4. Materials and Methods

### 4.1. Chemical and Reagents

Fetal bovine serum (FBS), Roswell Park Memorial Institute (RPMI)-1640 and penicillin-streptomycin were purchased from Gibco BRL Company (Grand Island, NY, USA). Modified Radioimmunoprecipitation assay (RIPA) lysis buffer, protease inhibitor cocktail, Commasie Plus™ Protein Assay Reagent, and chemiluminescent immunoblotting reagent were obtained from Thermo Fisher Scientific (Rockford, IL, USA). Syringic acid, pyrogallol, *p*-coumaric acid, catechin, gallic acid, protocatechuic acid, chlorogenic acid, caffeic acid, vanillic acid, ferulic acid, and ellagic acid, Sulforhodamine B (SRB) reagent, mitochondria membrane potential kit (JC-10 dye), propidium iodide (PI) and anti-*β*-actin antibody were obtained from Sigma-Aldrich (St. Louis, MO, USA). Primary antibody of PARP-1 was purchased from Santa Cruz Biotechnology (Dallas, TX, USA). Primary antibodies of cyclin D1, cyclin E, CDK-4, CDK-6, cleaved-caspase3, phospho-GSK-3*β*, GSK-3*β* (Ser9), phospho-*β*-catenin, *β*-catenin, phospho-Akt, Akt, and horseradish peroxidase-conjugated anti-mouse or rabbit-IgG were purchased from Cell Signaling Technology (Beverly, MA, USA). Apoptosis assay kits were obtained from Bio-Legend (San Diego, CA, USA).

### 4.2. Herb Materials

*Spirogyra neglecta* was harvested in 2018 from a local farm, Ban Nakhuha, Phrae Province, Thailand. The voucher specimen numbers of *S. neglecta* (No. 023242) were certified by the herbarium at the Flora of Thailand, Faculty of Pharmacy, Chiang Mai University and a specimen was kept for future reference.

### 4.3. Preparation of Spirogyra Neglecta Extracts and Fractions

Initially, 4 Liters of 80% (*v*/*v*) ethanol was mixed with 100 g of dried algae overnight. After filtration, the extract was evaporated with a rotary vacuum evaporator (BUCHI, Switzerland) in order to obtain ethanolic fractions. The ethanolic fractions were then freeze-dried to obtain an *S. neglecta* ethanolic extract (SnEE) powder. For the solvent partition purification step, the 10 g of *S. neglecta* ethanolic extract was dissolved in 350 mL of deionized water and subsequently partitioned with 500 mL of hexane. The hexane fraction was then separated and evaporated. Afterwards, the water fraction was partitioned with 500 mL of dichloromethane. After that, the dichloromethane fraction was collected, evaporated, and lyophilized. The water fraction was then partitioned again with 500 mL of ethyl acetate. The ethyl acetate fraction was collected, evaporated, and lyophilized. Finally, the residual fraction was lyophilized. Each fraction was kept at −20 °C for further experimentation.

### 4.4. Total Phenolic Content

The total phenolic content of herbal extracts used in this study was determined by the modified Folin–Ciocalteu assay as previously described [30,31], using gallic acid as a gold standard. Briefly, 0.4 mL of each concentration of the *S.neglecta* extracts was mixed with 0.3 mL of Folin–Ciocalteau reagent and kept in the dark at room temperature for 3 min. Then, sodium carbonate (Na_2_CO_3_) (0.3 mL) was added to the mixture, and it was further incubated in the dark at room temperature for an additional 30 min. The absorbance of the mixture was evaluated at 765 nm using a UV-visible spectrophotometer (UV-1800, SHIMADZU CO., LTD, Kyoto, Japan) and then compared to a standard curve that had been prepared with various concentrations of the phenolic compound commonly used for this assay, gallic acid (GA), in distilled water. The total phenolic content was shown as milligrams of GA equivalents per gram of the herbal extracts (mg GA/g extract).

### 4.5. Phenolics Content Determination Using HPLC

The HPLC analysis of *S. neglecta* ethyl acetate fraction (SnEA) was performed on a HPLC (Agilent Tecnologies, Santa Clara, CA, USA) using a reversed-phase C18 (250 mm × 4.6 mm, 5 µm) column. The mobile phase was composed of mobile phase A (0.1% trifluoroacetic acid) and mobile phase B (100% methanol) in gradient condition. The HPLC condition was modified from the previously described protocol [67]. The pre-injection time was set as 100%A: 0%B. The gradient elution was set as follows: 0–50 min, from 100 to 5% of A; 50.01–55 min stable at 5% of A, and 55.01–60 min from 5 to 100% of A. The detection wavelengths were 260 and 280 nm. The flow rate was set at 1.0 mL/min. The samples were run for 60 min. The quantitative data of bioactive compounds in SnEA were calculated from the respective calibration curves for standard caffeic acid, catechin, chlorogenic acid, ellagic acid, ferulic acid, gallic acid, *p*-coumaric acid, protocatechuic acid, pyrogallol, syringic acid, and vanillic acid to obtain the concentration of all the compounds (mg/g extract).

### 4.6. Cell Cultures

DU145 cells and PC3 cells, which are CRPC cell phenotypes, were purchased from ATCC (Manassas, VA, USA). The normal medium with fetal bovine serum (FBS) was used in this study. The cells were cultured in RPMI-1640 with 10% FBS, 50 IU/mL penicillin, and 50 g/mL streptomycin. The cells were maintained in a humidified incubator with an atmosphere comprised of 95% air and 5% CO_2_ at 37 °C. When the cells reached 70–80% confluency, the cells were harvested and plated for subsequent experimentation.

Primary human dermal fibroblasts (HDF) cells were aseptically isolated from an abdominal scar after a surgical procedure involving a cesarean delivery at the surgical operation room of Chiang Mai Maharaj Hospital, Chiang Mai University, Chiang Mai, Thailand (Study code: BIO-2558-03549 approved by Medical Research Ethics Committee, Chiang Mai University). The fibroblast cells were isolated as previously described [68]. The fibroblasts were cultured in DMEM supplemented with 10% FBS, 2 mM L-glutamine, 50 U/mL penicillin, and 50 μg/mL streptomycin. Cells were maintained in a 5% CO_2_ humidified incubator at 37 °C.

### 4.7. The Cell Viability Assay (SRB Assay)

The cytotoxicity of SnEA and its major compounds, pyrogallol, syringic acid and *p*-coumaric acid, against CRPC cells was measured using a sulforhodamine B (SRB) assay as was previously described. Briefly, DU145 and PC3 cells (5 × 10^3^ cells/well) were seeded in a 96-well plate and incubated at 37 °C, 5% CO_2_ overnight. After that, the cells were treated with or without various concentrations of SnEA (0–1000 μg/mL), syringic acid (0–500 μM; MW = 198.17), *p*-coumaric acid (0–600 μM; MW = 164.16), and pyrogallol (0–200 μM; MW = 126.11) for 24, 48, and 72 h. HDF cells (4 × 10^3^ cells/well) were seeded in a 96-well plate and incubated at 37 °C, 5% CO_2_ overnight. After that, the cells were treated with or without various concentrations of SnEA (0–1000 μg/mL) and pyrogallol (0–800 μM) for 48 h.

After incubation, 10% (*w*/*v*) trichloroacetic acid (TCA) was added to the cells and the cells were then incubated at 4 °C for 1 h. The medium was removed, and the cells were rinsed with slow-running tap water. Then, 0.054% (*w*/*v*) SRB solution (100 μL) was added to each well and the cells were incubated for 30 min at room temperature. The SRB solution was removed and then the cells were washed 4 times with 1% (*v*/*v*) acetic acid, and they were allowed to dry at room temperature. The dye was dissolved with 150 μL of 10 mM tris-based solution (pH 10.5) and the absorbance was measured at 510 nm using a microplate reader. The cells’ viability was calculated compared to the control.

### 4.8. Red Blood Cells (RBCs) Hemolysis

To determine the effects of SnEA and pyrogallol on human red blood cells (RBCs), a hemolysis induction assay was performed according to a previously described protocol [67]. Human blood samples were obtained from the blood bank laboratory Maharaj Hospital, Chiang Mai, Thailand which remained unused in the laboratory; for these samples the donor cannot be identified. Briefly, packed RBCs were diluted in 0.9% Normal saline solution (NSS) to give a 5% RBC suspension. The 300 μL of the 5% RBC suspension was incubated with or without various concentrations (0–40 μg/mL) of SnEA and pyrogallol (0–64 μM) in a 37 °C water bath for 4 h. NSS and 0.1% triton-X 100 were used as negative and positive controls, respectively. After 3 h, the supernatant was collected by centrifugation at 4400 rpm for 5 min at room temperature and hemoglobin concentrations were measured by spectrophotometry at 540 nm. The percentage hemolysis was calculated compared to positive control.

### 4.9. Mitochondrial Membrane Potential Assay

To determine the mitochondrial membrane potential using the mitochondrial membrane potential kit (JC-10 dye) (Sigma-Aldrich, St. Louis, MO, USA), the cells (4 × 10^3^ cells per well) were plated in 96-well black plate and were incubated overnight. The cells were treated with varies dose of SnEA or pyrogallol and incubated for 48 h. After incubation, 100 µL of treated media was removed and added to 50 µL per well of JC-10 dye diluted in assay buffer A, followed by incubation at 37 °C in a CO_2_ incubator for 30 min. Then, 500 µL of assay buffer B were added. The fluorescence intensity (λex = 490/λem = 525 nm) and (λex = 540/λem = 590 nm) was monitored for ratio analysis. The ratio of red/green fluorescence intensity is used to determine mitochondrial membrane potential.

### 4.10. Cell Cycle Assay

To determine the expression of cell cycle distribution, the cells (1 × 10^6^ cells per well) were plated in a 6-well plate and starved with 0.5% FBS in RPMI for 18 h before being treated with various doses of SnEA or pyrogallol. The cells were incubated with SnEA or pyrogallol for 24 h before harvested. The cell pellets were washed 2 times with PBS and were fixed with 70% methanol at −20 °C overnight. Then, the cell pellets were washed with PBS and 12.5 µL of ribonuclease A was added, followed by incubation at 37 °C in a CO_2_ incubator for 15 min. After that, 200 µL of propidium iodide (PI) was added, followed by incubation at 37 °C in a CO_2_ incubator for 45 min. The PI dye was removed by centrifugation and resuspended with 500 µL of PBS before being analyzed by flow cytometry (Beckman Coulter Inc., Indiana, USA).

### 4.11. Apoptosis Assay

The cells (1 × 10^6^ cells per well) were plated in 6-well plate and were treated with various concentrations of SnEA or pyrogallol for 24 and 48 h before being harvested. The cell pellets were washed 2 times with PBS, then stained with 5 µL annexin V-FITC and 10 µL propidium iodide (PI) using an apoptosis assay kit obtained from Bio-Legend (San Diego, CA, USA); they were kept for 15 min at room temperature. After incubation, 400 µL of reagent buffer was used before analysis by flow cytometry (Beckman Coulter Inc., Indianapolis, IN, USA).

### 4.12. Western Blot Analysis

To determine the expression of cell cycle regulator proteins (CDK-4, CDK-6, cyclin D1 and cyclin E), the cells (1 × 10^6^ cells per well) were plated in a 6-well plate and starved with 0.5% FBS in RPMI for 18 h; they were then treated with various doses of SnEA or pyrogallol. The cells were incubated with SnEA or pyrogallol for 24 h before harvesting. To determine the expression of pro-apoptotic and anti-apoptotic proteins, and the expression level of the Akt/GSK-3*β*/*β*-catenin signaling pathway in SnEA and pyrogallol treated CRPC cells, the cells (1 × 10^6^ cells per well) were plated in a 6-well plate and were treated with various doses of SnEA or pyrogallol for 24 or 48 h before harvesting. The cell pellets were lysed using RIPA buffer. Protein concentration was determined using the Bradford assay. The whole-cell lysate was subjected to 10% SDS-PAGE. The separated proteins were transferred into nitrocellulose membranes. The membranes were blocked with 5% of BSA in 0.5% TBS-Tween. After that, the membranes were washed twice with 0.5% TBS-Tween. Then, the membranes were further incubated overnight with the desired primary antibody at 4 °C. Next, the membranes were washed 4 times with 0.5% TBS-Tween followed by incubation at room temperature for 2 h with horseradish peroxidase-conjugated anti-mouse or rabbit-IgG depending on the primary antibody and were then washed 5 times with 0.5% TBS-Tween. Bound antibodies were detected using the chemiluminescent detection system and then exposed to X-ray film (GE Healthcare Ltd., Little Chalfont, UK). Equal values of protein loading were confirmed as each membrane was stripped and re-probed with an anti-*β*-actin antibody. Band density levels were analyzed using IMAGE J 1.410.

### 4.13. Statistical Analysis

All experiments were carried out in triplicate independent experiments to confirm reproducibility, and the data were presented as mean ± standard deviation (mean ± S.D.). Statistical analysis was carried out using Prism version 8.0 software. The independent *t*-test and one-way ANOVA with Dunnett’s test were used for data analysis from the following experiments: cell viability assay, cell cycle assay, apoptosis assay, mitochondrial membrane potential assay, Western blot assay. Significance was determined at * *p* < 0.05, ** *p* < 0.01 and *** *p* < 0.001.

## 5. Conclusions

Castration-resistant prostate cancer (CRPC) is an aggressive type of prostate cancer that is difficult to treat due to high proliferation and high metastasis rates. Accordingly, the discovery of novel anti-cancer compounds from medicinal herbs is essential to improve the treatment of CRPC patients. *S. neglecta* ethyl acetate fraction (SnEA) contained a high amount of a phenolic bioactive compound, pyrogallol, which displayed significant anti-cancer effects on CRPC cells (DU145 and PC3 cell lines). SnEA and pyrogallol downregulated the expression of cell cycle-regulated proteins and anti-apoptotic proteins, leading to cell cycle arrest at the G0/G1 phase and CRPC cells’ apoptosis. It was found that SnEA and pyrogallol could inhibit CRPC cell proliferation and promote cancer cell apoptosis via the regulation of the Akt/GSK-3*β*/*β*-catenin signaling pathway. The results obtained from this study emphasize the importance of the Akt and *β*-catenin pathway in CRPC in order to achieve targeted therapy. Moreover, *S. neglecta* extract and pyrogallol can potentially be used as an adjuvant therapy in the management of castration-resistant prostate cancer.

## Figures and Tables

**Table 1:** Total phenolic contents of *S. neglecta* extracts.

*Spirogyra neglecta* Extracts	Total Phenolic Content (mg GA/g Extract)
SnEE	245.73 ± 6.58
SnDM	37.68 ± 2.52
SnEA	735.42 ± 4.55 ***
Sn-H_2_O	27.33 ± 0.99

*** *p* < 0.001 vs. other *S. neglecta* extracts using independent *t*-test. Data are presented as mean ± S.D. values of three independent experiments.

**Table 2:** Identification of phenolic compounds in SnEA by HPLC technique.

Ethyl Acetate Fraction of *S. neglecta* (SnEA)	mg/g Extract (Mean ± S.D.)
Syringic acid	122.28 ± 11.72
Pyrogallol	107.53 ± 4.48
*p*-coumaric acid	93.22 ± 8.23
Catechin	10.11 ± 2.13
Gallic acid	9.96 ± 2.80
Caffeic acid	ND
Chlorogenic acid	ND
Ellagic acid	ND
Ferulic acid	ND
Protocatechuic acid	ND
Vanillic acid	ND

ND = Not detectable. Data are presented as mean ± S.D. values of three independent experiments.

**Table 3:** Selectivity index of SnEA and pyrogallol in the CRPC cell lines and human dermal fibroblast (HDF) cells.

	IC_50_ of SnEA (µg/mL) at 48 h	IC_50_ of Pyrogallol (µM) at 48 h
DU145 cells	47.94 ± 0.70	58.89 ± 3.06
PC3 cells	42.97 ± 4.24	45.79 ± 1.82
HDF cells	122.63 ± 2.25	201.68 ± 28.72
Selectivity Index (SI) * of SnEA and Pyrogallol Compounds		
HDF/DU145	2.55	3.42
HDF/PC3	2.85	4.40

* Selectivity index (SI) = IC_50_ of normal cells/IC_50_ of CRPC cells. SI > 2: indicated that the extract or compound has high selectivity for cancer cells; SI < 2: indicated poor selectivity for cancer cells.

## Data Availability

Not applicable.

## Supplementary Materials

The following supporting information can be downloaded at: https://www.mdpi.com/article/10.3390/ijms24076452/s1.
