# Supplementary material for: Pyrogallol from Spirogyra neglecta Inhibits Proliferation and Promotes Apoptosis in Castration-Resistant Prostate Cancer Cells via Modulating Akt/GSK-3β/β-catenin Signaling Pathway
Source: Int J Mol Sci. 2023 Mar 29;24(7):6452. doi: 10.3390/ijms24076452 (PMC10094533; doi:10.3390/ijms24076452)
Supplement: Supplementary file 1 [file ijms-24-06452-s001.zip › ijms-2290364-supplementary.pdf]

Supplementary material

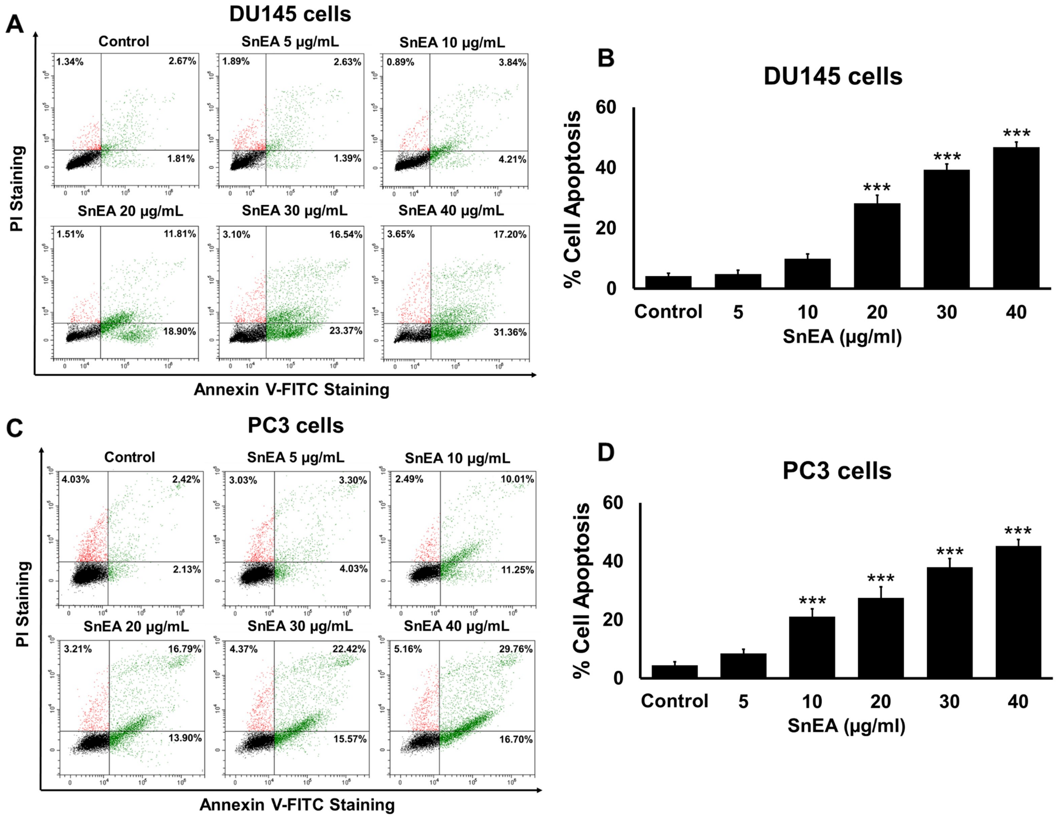

Figure S1: The effect of SnEA on DU145 and PC3 cells apoptosis. The DU145 (A, B) and PC3 (C, D) cells were treated with 0-40 μg/mL of SnEA for 24 hours before being co-stained with Annexin V-FITC and PI dyes. Cell death was analyzed by flow cytometry (A, C). The bar graphs show the percentages of apoptotic cells (B, D). Data are presented as mean ± S.D. values of three independent experiments. *** $p < 0.001$ vs. the control (0 μg/mL).

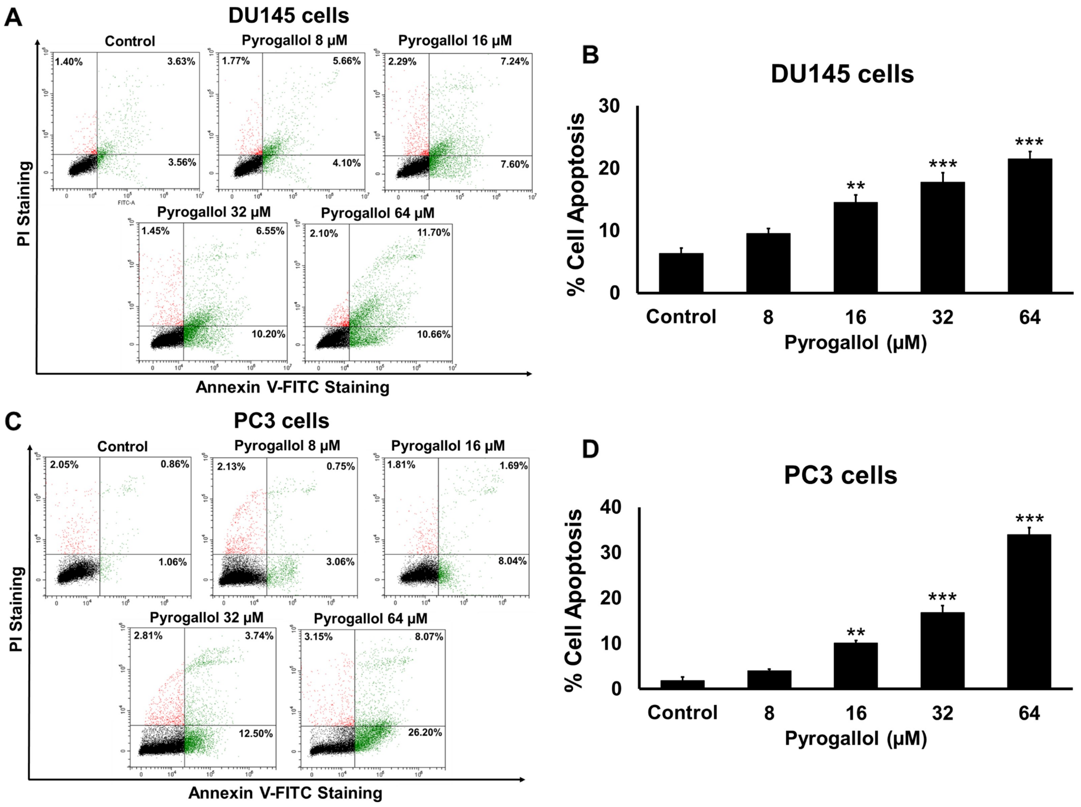

Figure S2: The effect of pyrogallol on DU145 and PC3 cells apoptosis. The DU145 (A, B) and PC3 (C, D) cells were treated with 0-64 μM of pyrogallol for 24 hours before being co-stained with Annexin V-FITC and PI dyes. Cell death was analyzed by flow cytometry (A, C). The bar graphs show the percentages of apoptotic cells (B, D). Data are presented as mean ± S.D. values of three independent experiments. ** $p < 0.01$ and *** $p < 0.001$ vs. the control (0 μM).
